# Detection of Left Atrial Enlargement Using a Convolutional Neural Network-Enabled Electrocardiogram

**DOI:** 10.3389/fcvm.2020.609976

**Published:** 2020-12-17

**Authors:** Junrong Jiang, Hai Deng, Yumei Xue, Hongtao Liao, Shulin Wu

**Affiliations:** ^1^School of Medicine, South China University of Technology, Guangzhou, China; ^2^Guangdong Cardiovascular Institute, Guangdong Provincial People's Hospital, Guangdong Academy of Medical Sciences, Guangzhou, China

**Keywords:** left atrial enlargement (LAE), convolutional neural network (CNN), electrocardiogram (ECG), echocardiography, artificial intelligence (AI)

## Abstract

**Background:** Left atrial enlargement (LAE) can independently predict the development of a variety of cardiovascular diseases.

**Objectives:** This study sought to develop an artificial intelligence approach for the detection of LAE based on 12-lead electrocardiography (ECG).

**Methods:** The study population came from an epidemiological survey of heart disease in Guangzhou. Elderly people (3,391) over 65 years old who had both 10-s 12 lead ECG and echocardiography were enrolled in this study. The left atrial (LA) anteroposterior diameter >40 mm on echocardiography was diagnosed as LAE, and the LA anteroposterior diameter was indexed by body surface area (BSA) to classify LAE into different degrees. A convolutional neural network (CNN) was trained and validated to detect LAE from normal ECGs. The performance of the model was evaluated by calculating the area under the curve (AUC), accuracy, sensitivity, specificity, and F1 score.

**Results:** In this study, gender, obesity, hypertension, and valvular heart disease seemed to be related to left atrial enlargement. The AI-enabled ECG identified LAE with an AUC of 0.949 (95% CI: 0.911–0.987). The sensitivity, specificity, accuracy, precision, and F1 score were 84.0%, 92.0%, 88.0%, 91.3%, and 0.875, respectively. Physicians identified LAE with sensitivity, specificity, accuracy, precision, and F1 scores of 38.0%, 84.0%, 61.0%, 70.4%, and 0.494, respectively. In classifying LAE in different degrees, the AUCs of identifying normal, mild LAE, and moderate-severe LAE ECGs were 0.942 (95% CI: 0.903–0.981), 0.951 (95% CI: 0.917–0.987), and 0.998 (95% CI: 0.996–1.00), respectively. The sensitivity, specificity, accuracy, positive predictive value, and F1 scores of diagnosing mild LAE were 82.0%, 92.0%, 88.7%, 89.1%, and 0.854, while the sensitivity, specificity, accuracy, positive predictive value, and F1 scores of diagnosing moderate-severe LAE were 98.0%, 84.0%, 88.7%, 96.1%, and 0.969, respectively.

**Conclusions:** An AI-enabled ECG acquired during sinus rhythm permits identification of individuals with a high likelihood of LAE. This model requires further refinement and external validation, but it may hold promise for LAE screening.

## Introduction

The left atrium (LA) is a crucial component of cardiac physiology, which is involved in collecting blood from pulmonary veins returning to the heart and regulating left ventricular filling during systole and diastole (1). Left atrial enlargement (LAE) can be caused by pressure overload and/or volume overload, which can lead to left atrial structural remodeling. Macrophages and neutrophils, as the key cellular mediators of inflammation, may also remodel the atria by infiltrating, releasing reactive oxygen species (ROS), and producing inflammatory cytokines and myeloperoxidases (2). LAE occurs commonly in association with diastolic dysfunction, left ventricular hypertrophy, mitral valve disease, and systemic hypertension (3, 4). There are no signs and symptoms of LAE in itself, and it is a pathophysiological response to other potential cardiovascular diseases. It has been proven that LAE can independently predict the development of a variety of cardiovascular diseases (5, 6). Although LAE can lead to the change of P-wave, the low sensitivity of ECG in diagnosing LAE limits its clinical application (7). Echocardiography, cardiac computed tomography (CCT), and cardiac magnetic resonance imaging (CMR) are the main means to diagnose LAE, and echocardiography is the most common choice because of its availability and safety (8, 9).

With the development of deep neural network models (DNNs), artificial intelligence (AI) has made great progress and has been gradually applied to the diagnosis of echocardiography and ECG (10, 11). Since DNNs can recognize patterns and learn useful features from raw input data without requiring extensive data preprocessing, feature engineering or handcrafted rules, and DNNs' performance tends to increase as the amount of training data increases, this approach is suitable for ECG analysis (12). AI-enabled ECG algorithm has achieved satisfactory results in the diagnosis of atrial fibrillation (AF), myocardial infarction (MI), and cardiac insufficiency (13–15), and its application in diagnosing LAE is also expected.

We hypothesized that in the absence of any severe heart disease, detection of LAE may be helpful in clinical decision making. There may be subtle changes in ECG due to the structural changes of LAE, and we can train DNNS to identify and diagnose LAE. Diagnosing LAE with this low-cost, convenient, and widely available method, we may screen out the potential cardiovascular diseases or the high-risk groups of specific cardiovascular diseases early.

## Methods

### Data Collecting

**Figure 1:**
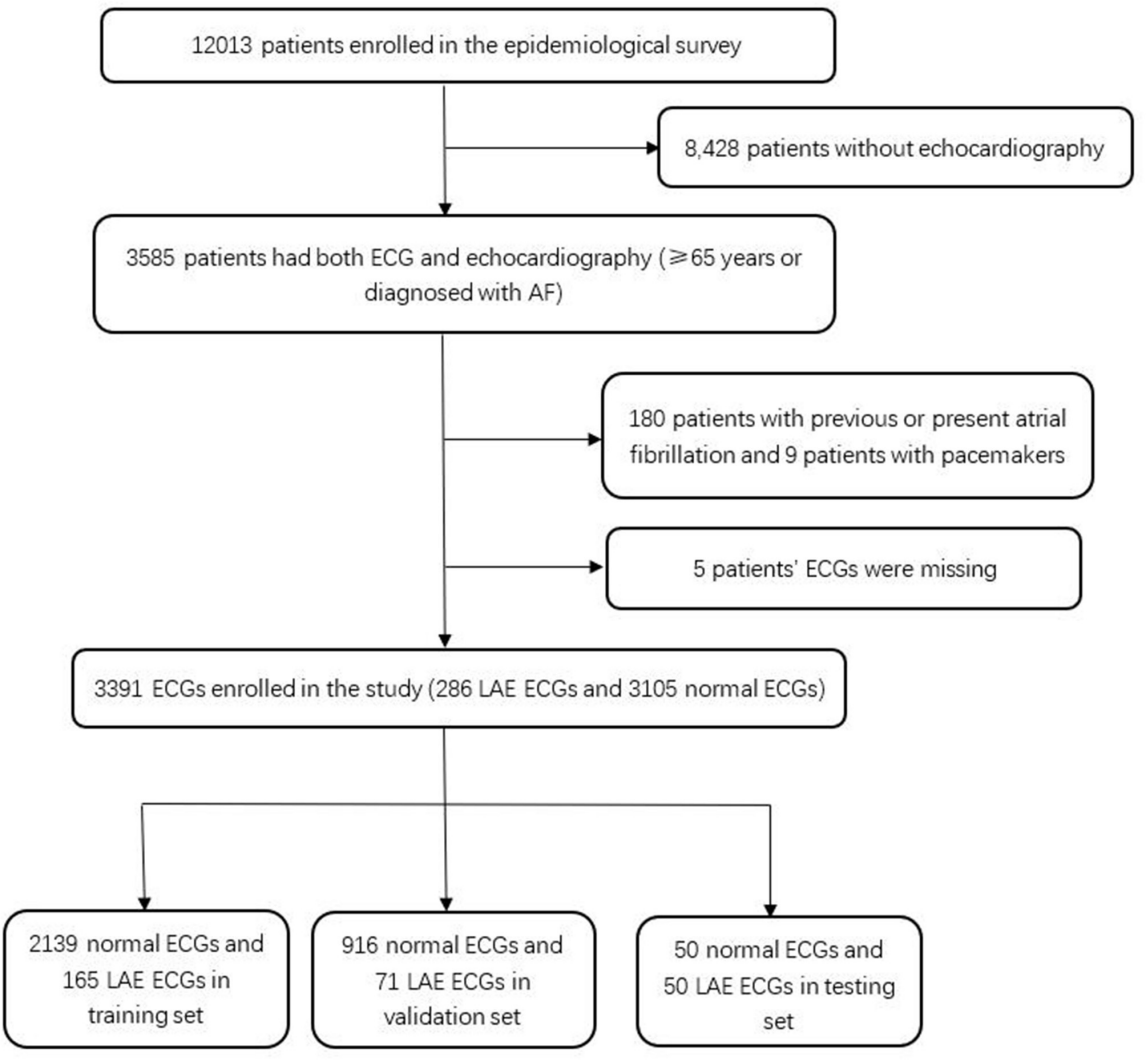
Patient flow diagram.

The study population came from an epidemiological survey of heart disease in Guangzhou, South of China. This survey was conducted from July 2015 to August 2017. Randomized multistage cluster sampling was used to recruit permanent residents aged 35 and above from Guangzhou City. More than 12,000 adults were enrolled in this survey, and those aged over 65 years old or diagnosed with AF (*N* = 3,585) underwent both standard 10-s, 12-lead, 500-Hz ECG, and echocardiography. The intervals between ECG and echocardiography were within 2 weeks, and the results were diagnosed and verified by two specialists. Patients (180) with previous or present atrial fibrillation and nine patients with pacemakers were excluded. Five patients' ECGs were missing, and the remaining 3,391 patients were all included in this study. The left atrial anteroposterior diameter >40 mm on echocardiography was diagnosed as LAE. ECGs were divided into the LAE group and the normal group. There were 286 ECGs in the LAE group, and 3,105 ECGs in the normal group (Figure 1).

### Data Preprocessing

**Figure 2:**
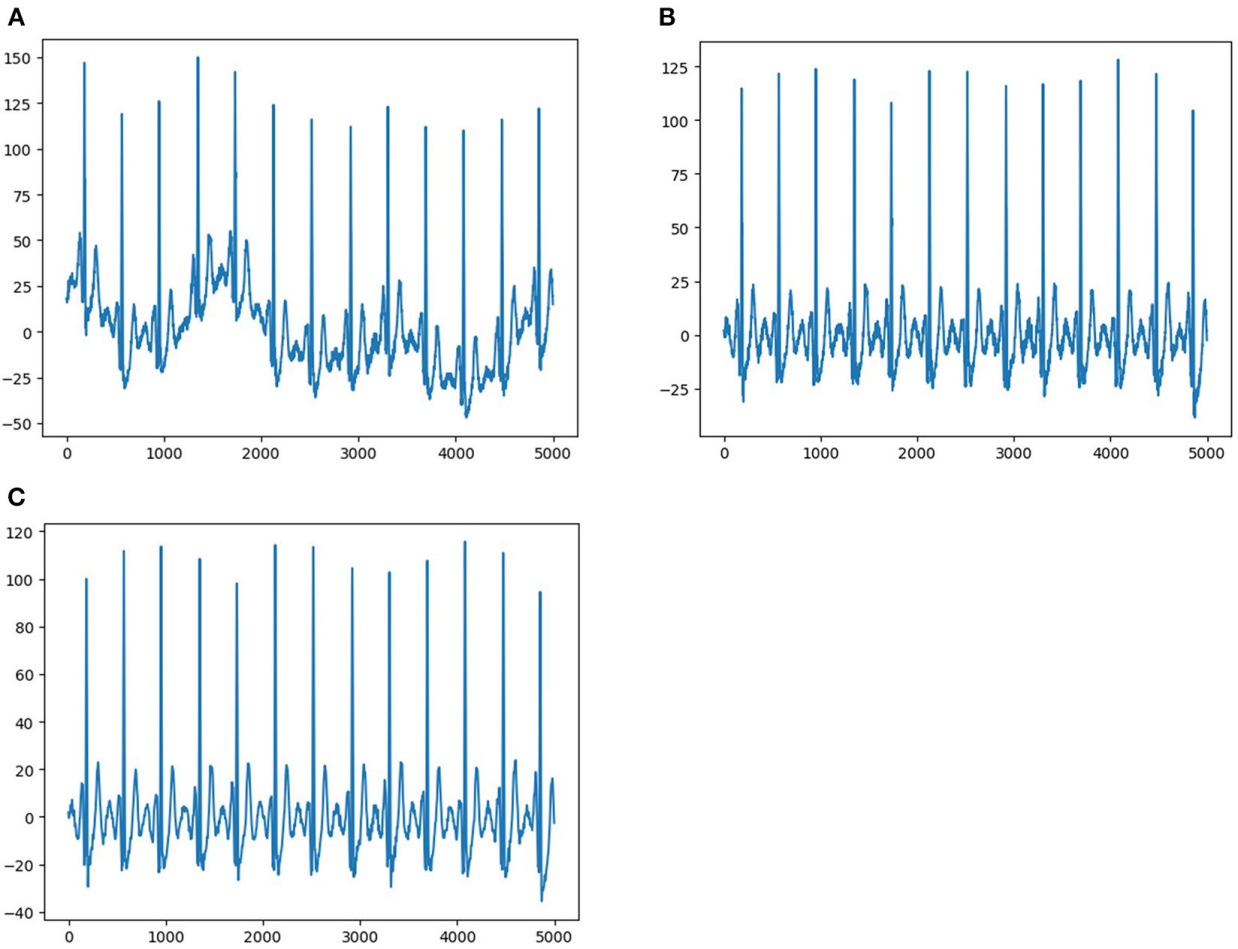
Data pre-processing. **(A)** Shows representative ECG with interference and baseline drift. **(B)** Shows representative ECG without baseline drift after preprocessing. **(C)** Shows representative ECG without interference and baseline drift after preprocessing.

Each ECG was a 12 × 5,000 (12 leads by 10-s duration sampled at 500 Hz) matrix, where the first dimension represented a spatial dimension, and the second represented a temporal one. The raw ECG data contained a large amount of noise and suffered from baseline drift (Figure 2A). Therefore, all raw ECG data were preprocessed before training. In order to eliminate the baseline drift and low power noise of raw ECG data, we first filtered the raw data using a low-pass filter to get the baseline and flattened the baseline by zeroing the mean (Figure 2B), and then we achieved denoising by filtering out the high-frequency signal (Figure 2C).

The 12-lead ECG is recorded using eight physical leads and four augmented leads created as a linear function of leads I and II, which do not contain incremental information. To optimize performance, we selected only the eight independent leads (leads I, II, and V1–6) because any linear function of the leads could be learned by the models. We used 8 s of ECG data by excluding the first and last 1-s periods because more artifacts were contained within these ranges. Consequently, we created two-dimensional (2D) data of 8 × 4,000 from each ECG to develop and validate the algorithm.

### Data Splitting

We randomly selected 50 ECGs from 3,105 normal ECGs and 50 ECGs from 286 LAE ECGs as the testing set, and then divided the left 3,291 ECGs into the training set and validation set at proportions of 7:3, respectively (Figure 1). In order to produce more data, we expanded the training and validation set by shifting the start point and choosing continuous 3,950 points of each lead, which contains 4,000 points in total, so it can be expanded to 50 samples for each sample by shifting the start point 50 times. After data expanding, the expanded training set contained 8,250 LAE samples and 106,950 normal samples, while the validation set contained 3,550 LAE samples and 45,800 normal samples. To avoid training bias caused by the imbalance of the training and validation data, we randomly sampled the same amounts of normal ECGs as the LAE ECGs for training. The training set was used to train the neural network, the validation set was used to optimize the network and select the parameters, and the testing set was used to evaluate the performance of the neural network. ECGs in different dataset were not repeated.

### Model Training and Developing

Convolutional neural networks (CNNs) were built by using the Keras Framework with a TensorFlow backend and Python. CNNs extracted the subtle changes in ECGs by convolution. Categorical cross entropy loss was used as the loss function, and Adam optimization method was applied. If the training process was not improved for 10 consecutive epochs, the training would be stopped. The training could be carried out in 100 epochs at most, and the minimum batch size was 64. A receiver-operating characteristic curve (ROC) was created for the testing and validation sets to assess the area under curve (AUC) of the AI-enabled ECG to determine whether LAE was present. Measures of diagnostic performance included the AUC, accuracy, precision, sensitivity, specificity, and F1 score. Multiple networks were tested, and the simplest (the one with fewer parameters or layers) that resulted in the highest AUC was selected. The selected network consisted of seven convolution layers. The first six layers were designed to learn features within each lead, the number of filters in the first convolution layer was 32, and the number of filters was doubled every two layers. The shapes of the filters were composed of 5 ^*^ 1 and 3 ^*^ 1 alternately. After each convolution layer, there was a “Relu” activation layer, a batch- normalization layer, and a max-pooling layer (4 ^*^ 1 after the first and forth layers and 2 ^*^ 1 after others). In the last convolution layer, the filter shape was 8 ^*^ 1, allowing it to fuse data from the different leads. After that, the data were fed to a dropout layer and a fully connected network with two hidden layers to avoid overfitting. The output layer had two classes and was activated using the “Softmax” function.

### Classification of Left Atrial Enlargement

LA anteroposterior diameter was indexed by body surface area (BSA) to quantify left atrial enlargement. According to the results, the data were divided into three groups: normal (<2.4 cm/m^2^), mild enlargement (≥2.4 and <2.7 cm/m^2^), and moderate–severe enlargement (≥2.7 cm/m^2^). There are 2,664, 550, and 177 patients in the normal, mild, and moderate–severe groups, respectively. Fifty ECGs were randomly selected from each group as the testing set, and then the left ECGs were divided into the training set and validation set at proportions of 7:3, respectively. Then the same model was trained to validate its ability to detect LAE in different degrees.

## Results

### Baseline Characteristics

The differences of clinical characteristics between the LAE group and the normal group are shown in Table 1. There are 139 males and 147 females in the LAE group with an average age of 72.6 years, while there were 1,135 males and 1,970 females in the normal group with an average age of 71.8 years. There were more males in the LAE group (*P* < 0.0001), and no differences were shown in age between the two groups (*P* = 0.0651). Moreover, hypertension, diabetes, myocardial infarction, valvular heart disease, cardiac insufficiency, obesity, and chronic kidney disease were more common in the LAE group (*P* < 0.05). Multivariate analysis further indicated that gender, obesity, hypertension, and valvular heart disease might be associated with LAE. Although the prevalence rates of myocardial infarction (*P* = 0.0545) and cardiac insufficiency (*P* = 0.0596) in the LAE group seemed to be higher, the statistical differences were still not reached.

**Table 1:** Clinical characteristics of patients.

		**LAE**	**Normal**	***P*****-Value**	
				**Univariate analysis**	**Multivariate analysis**
Age (mean)		72.6	71.8	0.0651	0.3670
Gender	Male	139	1,135	<0.0001	<0.0001
	Female	147	1,970		
BMI	<18.5	2	182	<0.0001	<0.0001
	18.5–25	92	1,914		
	25–30	147	881		
	≥87	45	128		
Hypertension	Yes	206	1,539	<0.001	<0.0001
	No	80	1,566		
Diabetes	Yes	54	436	0.0266	0.6911
	No	232	2,669		
Hyperlipidemia	Yes	76	781	0.5969	–
	No	210	2,324		
Myocardial infarction	Yes	24	117	0.0003	0.0545
	No	262	2,988		
Stroke	Yes	14	109	0.2330	–
	No	272	2,996		
Chronic kidney	<60	86	726	0.0115	0.8321
disease	≥32	200	2,379		
Valvular heart	No	180	2,555	<0.0001	<0.0001
disease	AVD	39	386		
	Mi-VD	40	111		
	Mu-VD	27	53		
EF (%)	<50	5	6	<0.0001	0.0596
	≥96	281	3,099		

*AVD, aortic valve disease; Mi-VD, mitral valve disease; Mu-VD, multiple valve disease*.

### Performance of AI Algorithm

**Figure 3:**
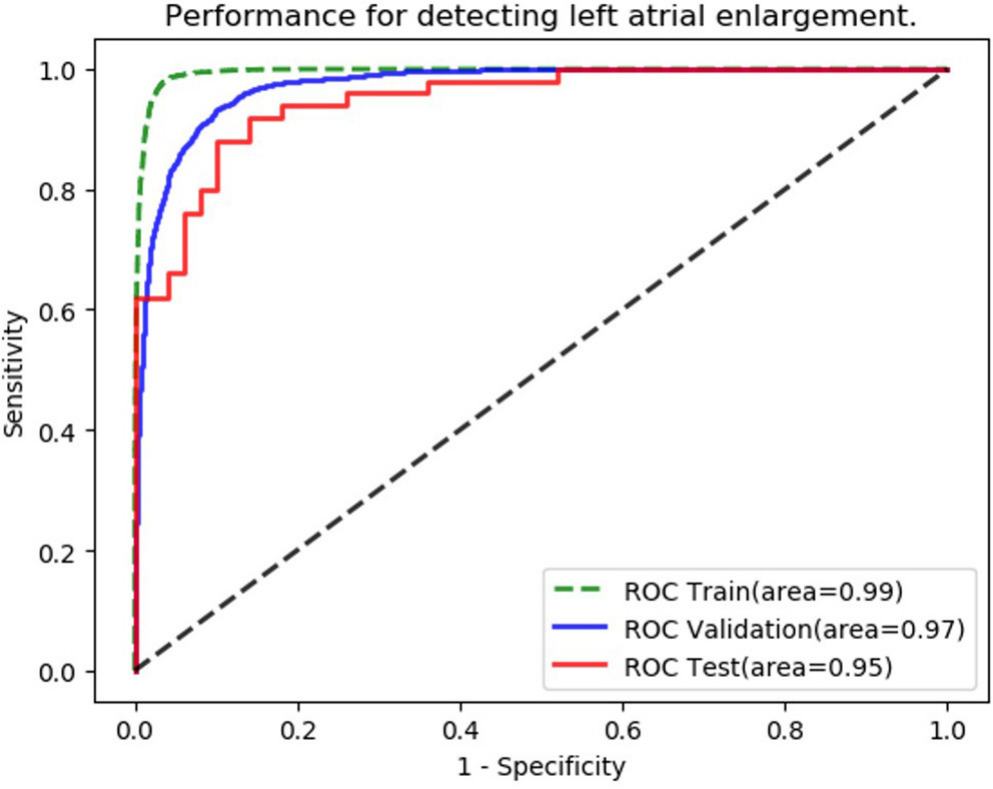
Performance of left atrial enlargement's (LAE's) diagnosis.

Following training and validation, the ROC curves of detecting LAE were drawn (Figure 3). The AUC of the validation set was 0.973 (95% CI: 0.969–0.976). 3,318 of 3,550 normal ECGs and 3,116 of 3,550 LAE ECGs were correctly diagnosed by the AI model, with sensitivity, specificity, accuracy, precision, and F1 scores of 87.8%, 93.5%, 90.6%, 93.1%, and 0.903, respectively (Table 2). The AUC of the testing set was 0.949 (95% CI: 0.911–0.987) with sensitivity, specificity, accuracy, precision, and F1 score of 84.0%, 92.0%, 88.0%, 91.3%, and 0.875, respectively. The results suggested that the AI model has a satisfactory ability to diagnose LAE. Compared with the AI model, only 42 normal ECGs and 19 LAE ECGs of the testing set were correctly diagnosed by physicians, with sensitivity, specificity, accuracy, precision, and F1 scores of 38.0%, 84.0%, 61.0%, 70.4%, and 0.494, respectively (Table 2).

**Table 2:** The confusion matrix of diagnosing left atrial enlargement (LAE).

			**Predicted**		**Sensitivity (%)**	**Specificity (%)**	**Accuracy (%)**	**Precision (%)**	**F1 scores**
			**Normal**	**LAE**					
Validation Set		Normal	3,318	232	87.8	93.5	90.6	93.1	0.903
		LAE	434	3,116					
Testing Set	AI	Normal	46	4	84.0	92.0	88.0	91.3	0.875
		LAE	8	42					
	Physicians	Normal	42	8	38.0	84.0	61.0	70.4	0.494
		LAE	31	19					

**Figure 4:**
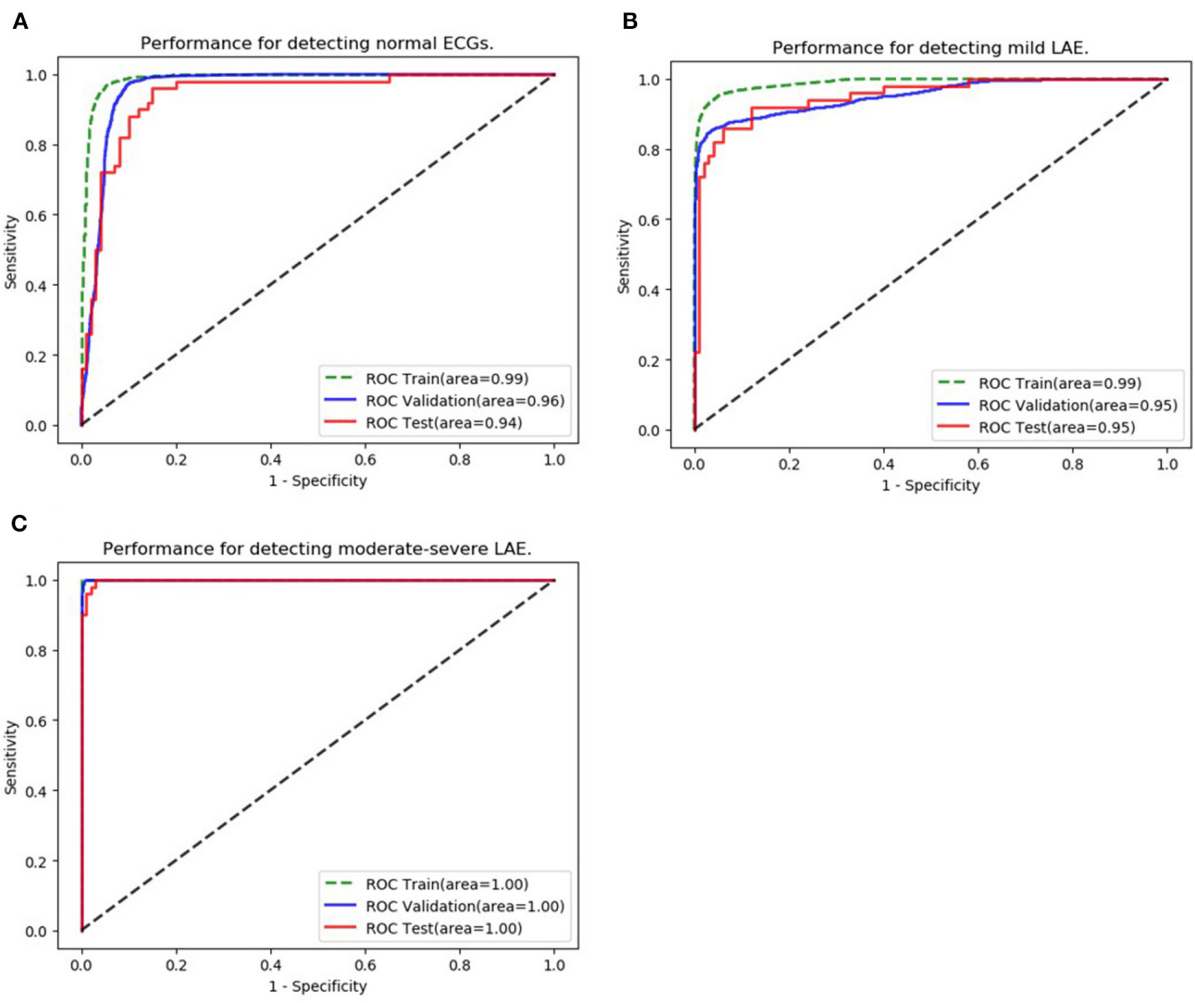
Classification of LAE. **(A–C)** Show the performance of the AI algorithm in classifying normal ECGs, mild LAE ECGs, and moderate-severe LAE ECGs.

The ability to classify LAE was also verified, ROCs were plotted for all classifications of LAE (normal, mild LAE, and moderate-severe LAE) (Figure 4). In the validation set, the AUCs of normal, mild LAE, and moderate-severe LAE were 0.962 (95% CI: 0.957–0.967), 0.953 (95% CI: 0.947–0.959), and 0.999 (95% CI: 0.999–1.00), while the AUCs in the testing set were 0.942 (95% CI: 0.903–0.981), 0.951 (95% CI: 0.917–0.987), and 0.998 (95% CI: 0.996–1.00), respectively. The confusion matrix of classifying LAE is shown in Table 3. The sensitivity, specificity, accuracy, positive predictive value, and F1 scores of diagnosing mild LAE were 82.0%, 92.0%, 88.7%, 89.1%, and 0.854, while the sensitivity, specificity, accuracy, positive predictive value, and F1 scores of diagnosing moderate-severe LAE were 98.0%, 84.0%, 88.7%, 96.1%, and 0.969, respectively. The CNN model achieved satisfactory results in classifying different degrees of LAE, especially in the diagnosis of moderate-severe LAE.

**Table 3:** The confusion matrix of classifying LAE by the artificial intelligence (AI) model.

		**Predicted**			**Sensitivity (%)**	**Specificity (%)**	**Accuracy (%)**	**Precision (%)**	**F1 scores**
		**Normal**	**Mild**	**Moderate-severe**					
Validation set	Normal	1,739	144	17	91.5	92.6	92.2	86.4	0.889
	Mild	265	1,628	7	85.7	95.5	92.2	91.7	0.886
	Moderate-severe	8	3	1,889	99.4	88.6	92.2	98.7	0.990
Testing set	Normal	43	5	2	86.0	90.0	88.7	81.1	0.835
	Mild	9	41	0	82.0	92.0	88.7	89.1	0.854
	Moderate-severe	1	0	49	98.0	84.0	88.7	96.1	0.969

## Discussion

In this study, we found that AI-enabled ECG performed well in diagnosing LAE (AUC 0.95), especially in diagnosing moderate and severe LAE. Compared with other medical screening tests, such as the B-type natriuretic peptide for heart failure (AUC 0.60–0.70) (16), Papanicolaou smear for cervical cancer (AUC 0.70) (17), and CHA2DS2-VASc Score for stroke risk (AUC 0.57–0.72) (18), the diagnostic performance was better.

LA size is an indicator for assessing the risk of cardiovascular disease (19–21). A clinical study involving 1,160 elderly patients suggested that LA size could independently predict cardiovascular events (22). Although most clinical studies on LA size have focused on the elderly, Leung et al. (23) found that LAE was also a predictor of multiple cardiovascular diseases through long-term follow-up of unselected young (mean age 47 years) patients with sinus rhythm (*n* = 483) (median follow-up of 6.8 years). Besides, LA size is also an important prognostic indicator for a variety of cardiovascular diseases. In patients with AF, LA size has predictive value for stroke risk, event-free survival, and recurrence after cardioversion (8). LAE has also been shown to predict the prognosis of myocardial infarction (24–26). In a clinical study of 314 patients with AMI followed up for 15 months, LAE was a powerful predictor of all-cause mortality (24). In addition, LAE is not only a predictor of prognosis in patients with dilated cardiomyopathy (DM) (27) and hypertrophic cardiomyopathy (HCM) (28, 29) but also has predictive value for the prognosis of mitral regurgitation or stenosis (8). Therefore, it is of great significance to identify LAE in a cheap, widely used, and convenient way. By screening the LAE population, we can identify the underlying cardiovascular disease causing LAE or screen out the high-risk population of specific cardiovascular diseases early and provide clinical guidance.

Echocardiography, as an effective means of detecting LAE, is cheaper and more available than cardiac computed tomography (CCT) and cardiac magnetic resonance (CMR), but it is still a high-cost screening tool for people at risk of cardiovascular disease. Therefore, a safe, convenient, low cost, and good performance detection method will be valuable as a new screening tool, especially in developing countries like China, where the level of primary care is relatively poor. Our data suggested that a simple, inexpensive, non-invasive 10-s 12-lead standard ECG may identify LAE patients with the aid of the AI algorithm.

Although the anteroposterior diameter may not exactly reflect the size of LA, especially in the case of asymmetric enlargement of LA, and LA volume has been described as a more accurate measure of LA size (30), the anteroposterior diameter is still a simple and acceptable measure of LA size that used in clinical studies (31, 32). LAE was diagnosed by the anteroposterior diameter of LA in this study because only the anteroposterior diameter was available in most participants. Since the size of LA increases with an increase in body size (5), LA anteroposterior diameter was indexed by body surface area (BSA) to quantify LAE. Identifying potential patients with LAE by AI-enabled ECG indicated that although no obvious abnormalities were observed in ECG, in fact, changes in cardiac electrophysiological signals caused by pathophysiological changes of the disease itself had already existed. Moreover, the greater the degree of LAE, the better the performance of artificial intelligence algorithms because with the degree of LAE increased, the model was easier to recognize the changes in cardiac electrophysiological signals. However, a key limitation in existing neural networks is interpretability. Decoding the “black box” will allow us to identify more abnormal electrophysiological signals in the ECG, and make physicians have a more accurate and comprehensive understanding of physiological and pathological ECG signals.

In this study, 286 of the 3,991 patients were diagnosed as LAE. The prevalence rate was 7.2%, and gender, obesity, hypertension, and valvular heart disease were related to LAE. Although the exact prevalence of LAE is not available, a study conducted by Bombelli et al. revealed that after over 10 years of follow-up, 123 of 1,045 patients (11.8%) with normal baseline LA size were progressed to LAE, and gender, obesity, hypertension may be related to LAE (33). Men and obese people have a larger LA, probably because they have a larger body size. In patients with myocardial infarction and/or systolic heart failure, the presence of LAE is common. However, myocardial infarction and cardiac insufficiency were not associated with LAE in this study. It may be because the prevalence of these two diseases were too low in the study population. LAE occurs commonly in association with mitral valve disease and systemic hypertension, which can lead to left atrial structural remodeling by pressure overload and volume overload. It is worth noting that, in patients with hypertension, LAE is likely to be substantially related to the presence and extent of left ventricular hypertrophy, as well as to the risk of development of hypertensive heart failure. Thus, detecting LAE with AI-enabled ECG will be of great value for the stratified management and treatment in patients with hypertension. In addition, the sensitivity, specificity, accuracy, precision, and F1 score of physicians in diagnosing LAE by ECG were 38.0%, 84.0%, 60.0%, 70.4%, and 0.494, respectively. Similar to this study, previous studies suggested that LAE detection by ECG had a poor sensitivity of 30–60%, and a high specificity of about 90% (7). The population included in this study was ≥65 years old. Although the AI algorithm performed well in this population, its diagnostic performance in younger population remained to be verified.

Although DNN performance tends to increase as the amount of training data increases, it is difficult to obtain such large amounts of labeled data in most circumstances. In fact, DNNs are also applicable in small datasets. Makimoto et al. (11) used the PTB ECG database consisting of 289 ECGs including 148 myocardial infarction (MI) cases to develop a CNN to recognize MI in ECG. The deep learning with a simple CNN for image analysis may achieve a comparable capability to physicians in recognizing MI on ECG. Using dermoscopic images of selected lesions from 514 patients, Phillips et al. developed an artificial intelligence algorithm to identify melanoma with an accuracy similar to that of specialists (34). Moving the start point of each lead to produce more samples may be helpful to the development of AI applications for ECG diagnosis with small datasets (35).

In conclusion, an AI-enabled ECG acquired during sinus rhythm permits identification of individuals with a high likelihood of LAE. This result could have important implications for screening for potential cardiovascular diseases that cause LAE or for high-risk groups of specific cardiovascular diseases. This model requires further refinement and external validation, but it may hold promise for LAE screening.

## Data Availability Statement

The original contributions presented in the study are included in the article/Supplementary Materials, further inquiries can be directed to the corresponding author/s.

## Ethics Statement

Written informed consent was obtained from the individual(s) for the publication of any potentially identifiable images or data included in this article.

## Author Contributions

JJ and SW designed the study. JJ developed the neural network. JJ and YX did the statistical analysis. JJ and SW wrote the manuscript. JJ, SW, YX, HD, and HL critically reviewed the manuscript. All authors contributed to the article and approved the submitted version.

## Conflict of Interest

The authors declare that the research was conducted in the absence of any commercial or financial relationships that could be construed as a potential conflict of interest.
